# Petrophysical and petrographic characteristics of Barail Sandstone of the Surma Basin, Bangladesh

**DOI:** 10.1007/s13202-021-01196-0

**Published:** 2021-05-31

**Authors:** Md Shofiqul Islam, Md Hasibul Hasan Shijan, Md Samin Saif, Pradip Kumar Biswas, Muhammad Omar Faruk

**Affiliations:** 1 Department of Petroleum and Mining Engineering, Shahjalal University of Science and Technology, Sylhet, 3114 Bangladesh; 2 Marine Geology and Geophysics, Lamont Doherty Earth Observatory, Columbia University, Palisades, NY 10964 USA; 3 Bangladesh Council of Scientific Research, Institute of Mining, Mineralogy and Metallurgy, Khanjanpur, Joypurhat, 5900 Bangladesh; 4 Department of Physics, Shahjalal University of Science and Technology, Sylhet, 3114 Bangladesh

**Keywords:** Barail sandstone, Petrography, Petrophysical properties, Reservoir rock, Porosity, Permeability

## Abstract

The Barail sandstone in the Surma Basin is a medium- to coarse-grained pinkish-colored rock exposed near the northeastern margin of Bangladesh. In this study, we evaluated the reservoir quality of the Barail sandstone based on its petrophysical and petrographic characteristics. Petrophysical analyses of outcropped samples showed that sandstones are made up of 16.48% porosity and 132.48 mD permeability. Sandstone density ranges from 1.94 g/cm3 to 2.37 g/cm3, with a mean value of 2.12 g/cm3, shown as moderately compacted sandstone. Integrated data such as bulk density, porosity, permeability, Rock Quality Index (RQI), Normalized Porosity Index (NPI), Flow Zone Indicator (FZI), compressive strength, etc. with their relationships indicate that Barail sandstone owing characters to become a good petroleum reservoir. The rock samples consisted mainly of quartz with an insignificant amount of rock fragments and plagioclase feldspar and are categorized as sub-arkose to sub-litharenite. The rock samples also contains lithic (andesine, microcline, muscovite, biotite, etc.) of granitic and gneissic fabric and some volcanic product like aguite, albite, andesine, garnet, spinel and ulvo-spinel indicating the source of nearby orogeny. The euhedral to subhedral shape of the quartz grain in a porphyritic texture, moderately sorted with a smaller amount of clay minerals indicating the moderately mature rock type. The iron oxide border around the quartz grain also indicates that the Barail sandstone was deposited under dry climatic condition.

## Introduction

Petrophysical analysis involves the investigation of physical and chemical properties and their interaction with fluids (Tiab and Donaldson 2004). The most important properties of petrophysical analysis are lithology, porosity, permeability, water saturation, resistance and density. These parameters can be evaluated using core samples and geophysical measurements (seismic and well records). Geological, geophysical and reservoir engineering studies provide a complete image of the underground reservoir. Petrophysical properties help engineers and geoscientists understand the rock properties of the reservoir, especially the way subsurface pores are interconnected, controlling the accumulation and migration of oil.

Sedimentary rocks, particularly sandstone and limestone, are the most common reservoir rocks, since they are more porous and permeable than most igneous and metamorphic rocks. The Surma Group sandstone is considered reservoir rock in Bangladesh because all oil/gas production comes from it (Bokabil and Bhuban Formation). The Bokabil Formation is sand-dominated thick channel deposits with finely interlaced shale and siltstone with porosity ranging from 10 to 20% (Alam et al. 2003).

The maximum borehole depth is 4970 m in the Atgram structure in the Bhuban Formation with good reservoir quality. Under the Surma Group, the Oligocene Barail sandstone of medium to coarse grain might be prospective reservoir rocks, still to be analyzed. Barail sandstone is well exposed in the Upper Assam (Sen et al. 2012) and Mizoram (Borgohain et al. 2020; Hauhnar et al. 2021) regions. Barail sandstone is mainly poor to moderate sorted, sub-arkose to sub-litharenite type with abundance of silica (Borgohain et al. 2020; Hauhnar et al. 2021; Sen et al. 2012). Barail sandstone is moderately mature, grayish to reddish color and experienced medium to high intensity of chemical weathering in the parent rocks under humid climatic condition (Borgohain et al. 2020) to arid (Sen et al. 2012). Barail sandstone might be derived from active upper continental area (Sen et al. 2012; Borgohain et al. 2020) resembling to granitic-gneisses and were deposited in active continental margin (Sen et al. 2012). These sandstones are exposed close to the border between Bangladesh and India and appear to be over-thrusted in the Dauki Fault area. Sandstones are grey-white to pinkish in color with medium to coarse grains and interspersed with fine-grained silty clay.

Kopili shale has been studied (Jahan et al. 2017) to justify its potential as a source rock with a TOC value of ~ 1% just below the Barail sandstone (Table 1). Considering the source rocks (such as Kopili shale and Sylhet limestone), the Barail sandstone Formation is a potential reservoir rock if it has sufficient porosity and permeability. There have been no significant works done yet on the Barail sandstone especially for Bangladesh part. In this study, we characterize the outcropping Barail sandstone as a reservoir rock based on its petrophysical and petrographic properties. This study will help exploration activities in the Surma Basin to enhance the petroleum prospects for reservoir at greater depth.

**Table 1:** Stratigraphy of the shelf area in the Bengal Basin (modified after (Alam et al. 2003; Islam et al. 2021)

Age (approx.)	Group	Formation	Legend	Thickness (max., in meter)
Holocene	Dihing Dupi Tila	Alluvium		3350
Pleistocene		Dihing Upper Dupi Tila Lower Dupi Tila		
Mid-Pliocene	Tipam	Girujan Clay Tipam Standstone Upper Marine Shale Upper		3500
Early Pliocene Miocene	Surma			3900
Oligocene	Barail	Lower		
Paleocene-eocene	Jaintia	Kopili Sahle		7200
		Sylhet limestone		
		Tura sandstone		
Pre-paleocene	Undifferentiated sedimentary rocks (with some volcanics?) on the continental basement complex			

## Geologic setting of the study area

**Fig. 1:**
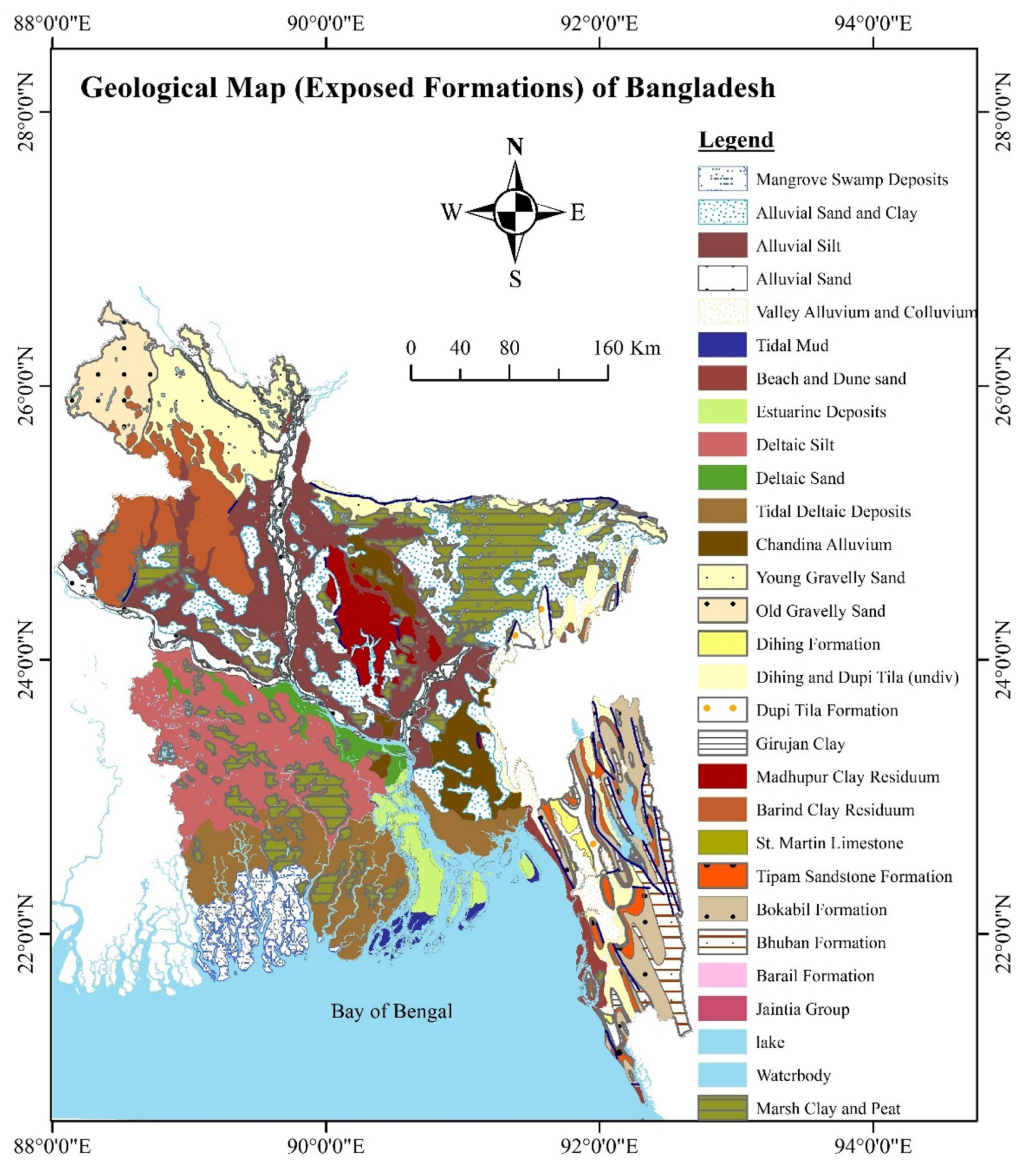
Geological map of Bangladesh modified after (Alam et al. 2001)

**Fig. 2:**
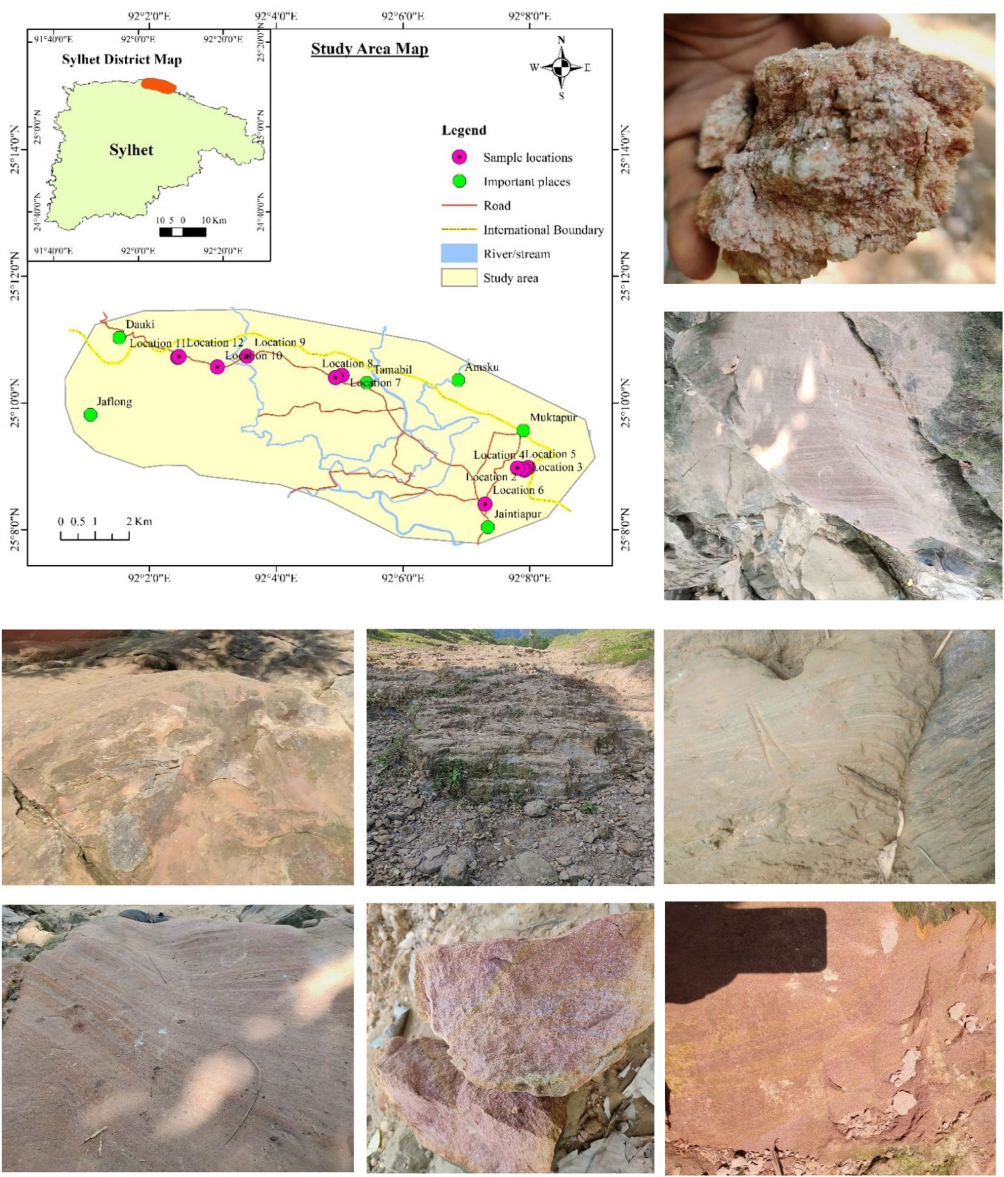
Map of study area and photographs of exposed Barail Sandstones

The study area is situated close to the international border of Bangladesh and India and at the northern margin of the Surma Basin, is an over-thrusted area of the E-W Dauki fault (Fig. 1). The Dauki Fault is north-dipping boundary fault of the Shillong Plateau (Bilham and England 2001; Islam et al. 2011) accommodating to significant regional stress that could cause an earthquake in the future. The Surma Basin is a tectonically active subsiding basin of a huge sedimentary cover of ~ 16 km (Mannan 2002). The basin is subsided mainly from Oligocene to Pliocene, and they are deposited almost exclusive typical sequences of tidal to fluvial, and to a lesser degree marine sandstone, siltstone, and shale (Table 1; Fig. 2). These sediments were subjected to subsequent phases of orogenesis of the Himalayan front, resulting in the formation of relatively younger folds of the folded frontal belt (FUR 2011). Geographically, the study area is bounded by longitude of 92°1´30´´E to 92°9´0´´E and latitude of 25°8´0´´ to 25°11´30´´ N (Fig. 2 (upper left)).

The eastern part of the Surma Basin is folded, and it is a part of Indo-Burma Folded Belt which is produced by eastward subduction of the Indian plate beneath Burmese sub-plate below the Bengal Basin (Steckler et al. 2016). This basin is a potential petroleum basin in the Southeast Asia with proven reserves in Miocene Sandstone (Bokabil Formation) and contains all elements of a petroleum system (Islam et al. 2015). Stratigraphically, the Barail sandstone (Oligocene), the Surma group (Miocene inferior to Miocene medium) and the Tipam sandstone (Miocene superior to Pliocene early) form the complete sediment succession with good reservoir characteristics. Bhuban Formations of the Surma Group have a thickness of about 5000 m and are further subdivided into upper, middle and lower units. The Bokabil Formation is dominated by sand and has very high-quality reservoir characteristics deposited into the terrestrial environment (Islam et al. 2015). Lithology of Surma Group rocks consisted of siltstone, shale sandstones, mudstone and various mixtures (Hauhnar et al. 2021).

The Surma Basin was formed structurally by the contemporaneous interaction of two major tectonic elements; the Pop uping the Shillong Plateau to the north and the westward moving mobile Indo-Burma Fold Belt. The tectonic movement is considered to have occurred from the Neogene to the present, with the strongest period of crustal disturbance during the middle Miocene. The main result of this tectonics is a series of asymmetric anticline oriented north–south in eastern Bangladesh, in which the degree of deformation increases eastward. Basin relief, structural elements, growth rate, trap style, source rocks and maturities are suitable for forming commercially sized gas-bearing structures (Fatta et al. 2018; FUR 2011).

## Method of study

Samples were collected from twelve (12) locations within exposed sections (Fig. 1b) in Jaintapur and Gowainghat Upazila, Sylhet District. Nineteen (19) core samples were prepared for petrophysical and petrographic analyses (Fig. 2).

### Petrophysical analyses

Major petrophysical analyses such as density, porosity, permeability and rock compressibility were measured at the room temperature and ambient pressure at the Petrophysical and Geotechnical Engineering Laboratories, Department of Petroleum and Mining Engineering (PME), Shahjalal University of Science and Technology (SUST). The cylindrical samples (about 3.7 cm × 6.5 cm) were prepared from collected samples from the field with help of core plug. The samples were cleaned and then dried at a temperature of 90 °C overnight to avoid deformation and alteration of clay by heating.

## Density measurement

The bulk density (*σ*_b_) of rock samples was measured using direct methods for geometric shapes (cylindrical plugs), where the bulk volume (*V*_b_ and dry weight of the core samples (*W*_d_) were measured using a precision caliper (0.1 mm precision) and an electronic balance (0.1 mg precision).

The measured rock bulk density (*σ*_b_) was calculated as:

1 $$\sigma_{b} = \frac{{W_{d} }}{{V_{b} }}$$

TPI-219 Helium porosimeter (Core test system Inc.) was used to determine the porosity. The porosity of the rock (Bilham and England) uses the two helium matrix-cup porosimeters for the estimation of grain volume, which follows Archie's law for determining bulk volume. Grain density (*σ*_g_) was determined by the product of the porosity measurement using the equation below.

2 $$\sigma_{g} = \frac{{W_{d} }}{{V_{g} }}$$

where *V*_g_ is the volume of rock grains, which can be calculated according to the following equation

3 $$V_{g} = V_{b} - V_{p}$$

where *V*_p_ is the volume of the pores.

Permeability was measured using TKA 209 gas permeameter. Permeability was measured by the following equation:

4 $$k = \frac{q\mu L}{{A\Delta P}}$$

where *k* is the permeability in mD, *Q* is the rate of flow, cm/sec, *μ* is the viscosity, centipoises, ΔP is the pressure gradient, atm/cm, *A* is the cross-sectional area, cm^2^ and L is the length of sample, cm.

Each distinct reservoir unit has a unique flow zone indicator (FZI), reservoir quality index (RQI) and normalized porosity index (NPI) values (Al-Dhafeeri and Nasr-El-Din 2007; Kassab and Teama 2018). The concept of (Amaefule et al. 1993) is based on the calculation of two terms, RQI and NPI, as defined below:

5 $${\text{RQI}} = 0.0314 \times \sqrt {\frac{k}{\emptyset }}$$

6 $${\text{NPI}} = \frac{\emptyset }{1 - \emptyset }$$

RQI and NPI are used to determine FZI, which is a unique and useful value to quantify the flow character of a reservoir and one that offers a relationship between petrophysical characteristics at small-scale, such as core plugs, and large scale, such as wellbore level (Al-Dhafeeri and Nasr-El-Din 2007).

7 $${\text{FZI}} = \frac{{{\text{RQI}}}}{{{\text{NPI}}}}$$

8 $${\text{Log}}\left( {{\text{RQI}}} \right) = {\text{Log}}\left( {{\text{FZI}}} \right) + {\text{Log}}\left( {{\text{NPI}}} \right)$$

where 0.0314 is constant, *k* is the permeability, *ϕ* is the effective porosity (%), NPI is the ratio of pore volume to grain volume and RQI and FZI are in microns.

## Rock compressibility measurement

**Fig. 3:**
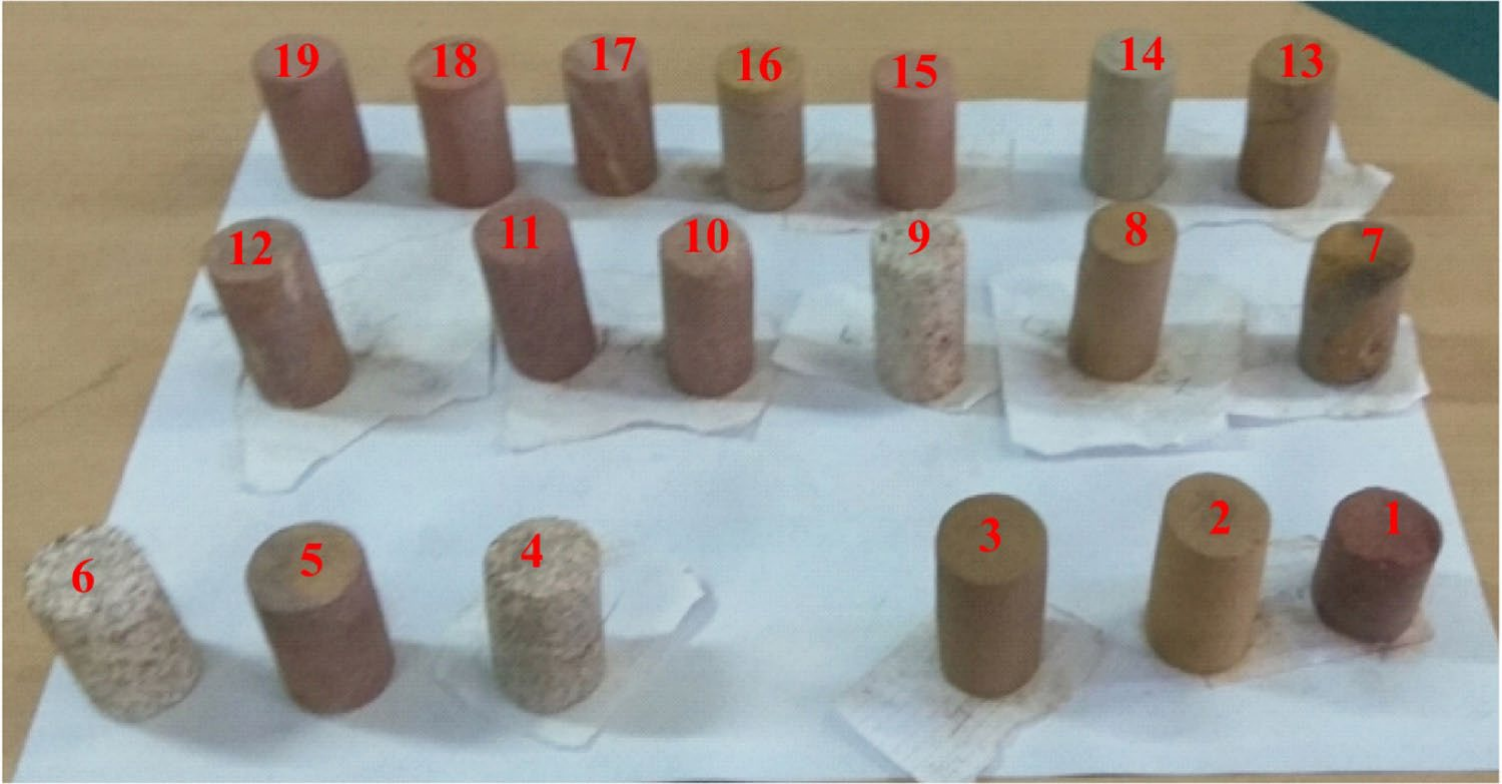
Samples prepared using core plug for petrophysical analyses

The compressibility of the rock (compressive stress, shear stress, Young modulus and Poisson ratio were measured using an open compression resistance test (in accordance with ASTM D 2166) using triaxial (Triax, Wykeham Farrance, Control Group) setup at the Geotechnical Engineering Lab, PME, SUST at a vertical speed of 0.2 mm/m. (Fig. 3).

## Petrographic measurement

**Fig. 4:**
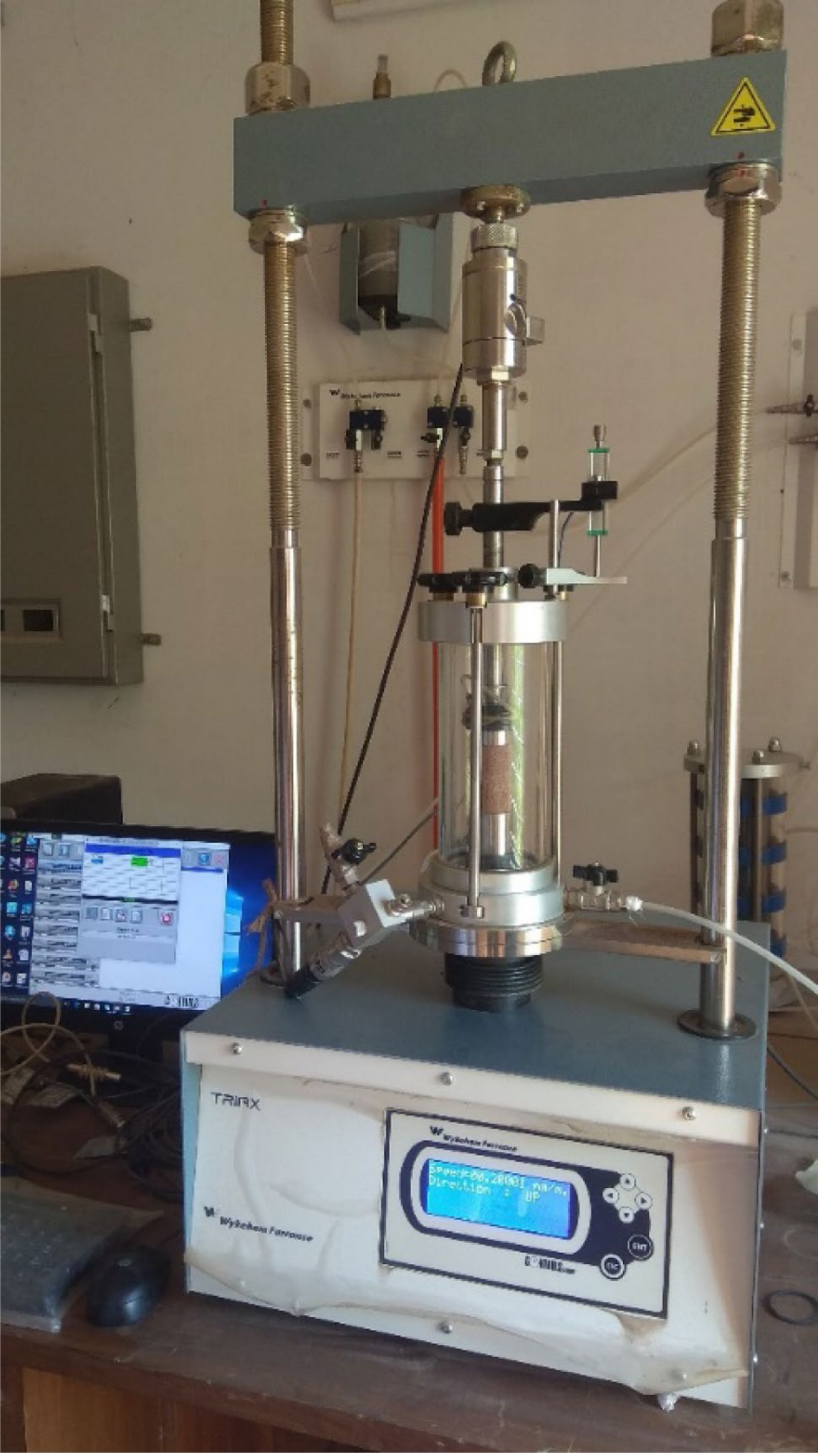
Photograph showing the tri-axial setting for unconfined compressive strength test

The specimens were examined with different types of microscopes. Thin sections were prepared and examined by an optical polarized transmitted light microscope device (Olympus BX51 TF Japan and OMAX-BMQB333V173 BDKC180L3) connected with a digital camera under 10X up to 40X magnification. X-ray diffraction (XRD) was carried out in the materials laboratory of the Department of Physics, SUST, for selected rock samples (six samples). XRD analyses for six samples were carried out using Rigaku Ultima-IV and were performed with Cu-Kβ radiation operating at 9 kV with D/teX Ultra 250 detector of a scan rate of 0.01° 2θ per second with scan range 15–80°. Mineral grains in sandstone and rocks fragments were identified using the WPPF software which uses Crystallographic Open Database (COD) as a baseline database. The COD could fully identify “small molecules/small to 180 medium unit cells” crystal configurations. Thin sections were prepared for all samples but selective samples were analyzed based on their quality (Fig. 4).

## Result

### Petrophysical characteristics

The petrophysical properties given in Table 2 clearly indicate that the Barail sandstones are very heterogeneous. The heterogeneity of the data presented is mainly attributed to differences in rock lithology, heterogeneity in crystal size and intergranular relationships.

**Table 2:** Petrophysical properties of Barail sandstone samples

Sample number	Sample location	Bulk rock Density (g/cm3)	Bulk grain density (g/cm3)	Porosity (%)	Permeability (mD)	Reservoir Quality Index (RQI)	Normalized porosity Index (NPI)	Flow zone indicator (FZI)	Compressive strength (kPa)	Shear strength (KPa)	Young's modulus (MPa)	Poisson's ratio
1	1	2.07	2.69	15.77	173.31	0.10	0.19	0.56	2456.2	924.78	1879.62	0.16
2	2	2.27	2.44	11.63	48.37	0.06	0.13	0.49	2715.4	698.24	3183.24	0.33
3	3	2.00	2.54	12.17	82.08	0.08	0.14	0.59	1898.9	562.54	2561.30	0.20
4	3	2.20	2.66	14.51	125.11	0.09	0.17	0.54	2564.3	601.25	2862.01	0.19
5	4	2.29	2.55	14.13	171.94	0.11	0.16	0.67	2896.4	670.23	2154.68	0.34
6	5	2.00	2.69	16.51	160.48	0.10	0.20	0.49	2168.8	584.00	2591.00	0.27
7	8	2.08	2.64	13.44	44.70	0.06	0.16	0.37	2323.3	875.27	3786.21	0.31
8	8	2.05	2.56	10.29	95.00	0.10	0.11	0.83	2056.9	625.38	1796.52	0.18
9	9	2.13	2.40	16.71	114.23	0.08	0.20	0.41	2174.2	587.00	2610.50	0.24
10	10	2.33	2.28	17.80	13.79	0.03	0.22	0.13	2132.6	536.86	2867.16	0.31
11	10	1.98	2.67	12.97	83.30	0.08	0.15	0.53	2125.6	756.53	1986.57	0.17
12	10	2.37	2.45	13.64	13.08	0.03	0.16	0.19	3426.2	568.95	2452.81	0.20
13	11	2.04	2.57	24.13	105.72	0.07	0.32	0.21	3157.9	579.00	1686.60	0.24
14	11	1.96	2.84	19.67	187.51	0.10	0.24	0.40	2167.7	584.00	2694.00	0.23
15	11	2.06	2.28	18.89	266.07	0.12	0.23	0.51	2175.7	679.00	2936.80	0.23
16	11	2.12	2.75	20.28	151.95	0.09	0.25	0.34	2154.7	577.00	3501.10	0.33
17	11	1.94	2.39	23.79	282.62	0.11	0.31	0.35	1835.6	653.49	1964.82	0.21
18	12	2.10	2.28	21.80	358.90	0.13	0.28	0.46	2303.6	552.00	1058.50	0.24
19	12	2.25	2.58	14.93	38.92	0.05	0.18	0.29	2920.2	760.08	4348.00	0.33
Avg		2.12	2.54	16.48	132.48	0.08	0.20	0.44	2402.8	651.35	2574.81	0.25

The petrographic and petrophysical characteristics of samples are grouped into two main facies: facies 1 (sub-arkose) and facies 2 (sub-litharenite).

Barail Sandstone has porosity ranges from 10.29 (for sample 8) to 23.79 (for sample 17) with an average value of 16.48 (Table 2). Owing of larger porosity (> 10%), these sandstones can be considered as a prospective reservoir rock in the Surma Basin. The permeability values of the analyzed samples have ranges from 13.08 (sample 12) to 358.90 (sample 18) with an average value of 132.48 (Table 2). Porosity and permeability of the Barail sandstone have linear relations that indicated a good reservoir quality. The bulk density values of the samples are ranges from 1.94 g/cm3 to 2.37 g/cm3 with an average value 2.12 g/cm3 (Table 2). RQI, NPI and FZI values also range from 0.03 to 0.13 (avg. 0.08), 0.11 to 0.32 (avg. 0.20) and 0.13 to 0.83 (avg. 0.44), respectively (Table 2). Compressive strength (rock strength) of the samples ranges from 536.86 Mpa to 924.78 Mpa with an average value of 651.35 MPa (Table 2).

**Fig. 5:**
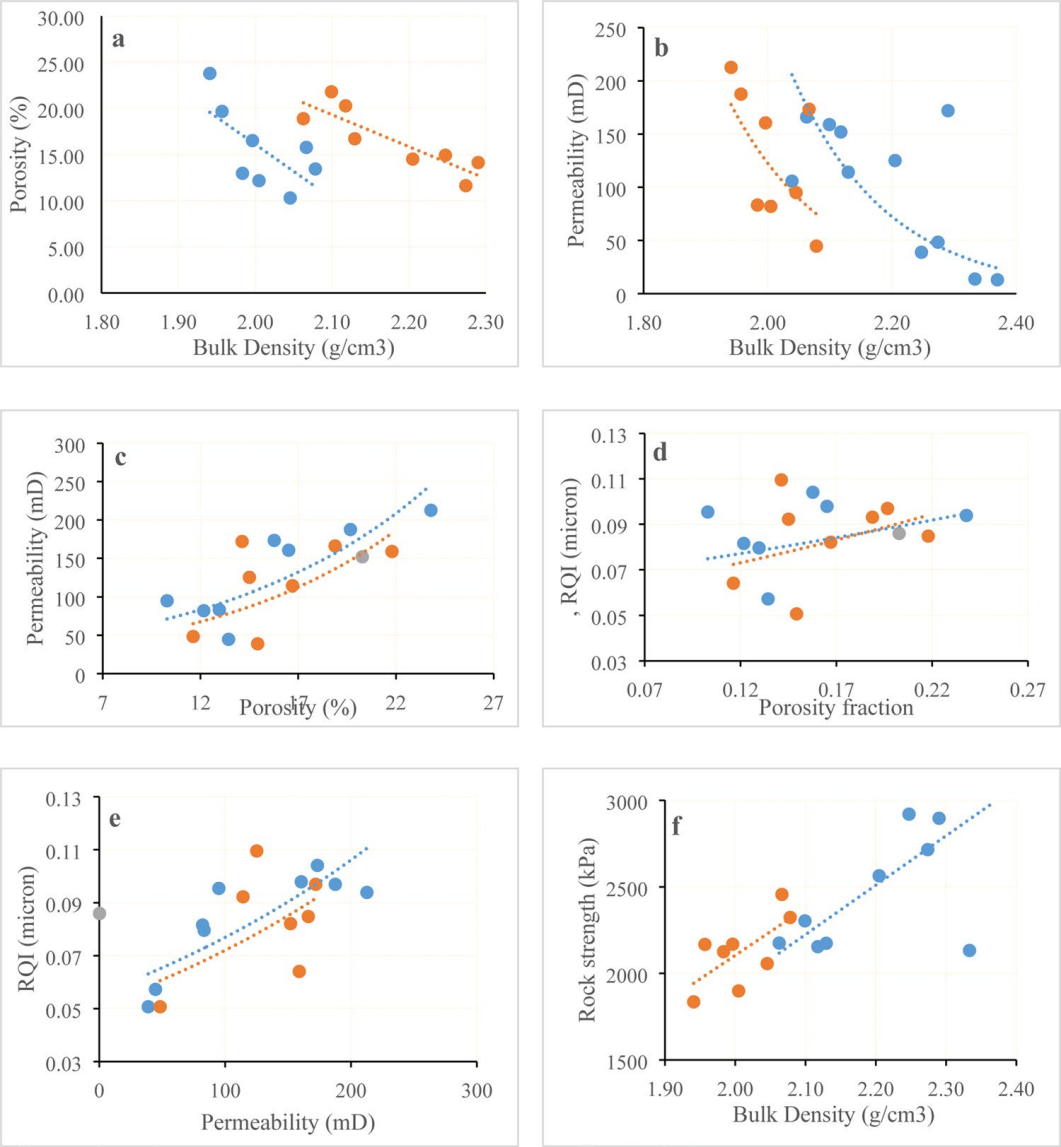
Cross-plots of **a** Porosity versus bulk density, **b** Permeability versus bulk density, **c** Porosity versus permeability, **d** RQI versus porosity fraction, **e** NPI versus porosity fraction and **f** Rock strength versus bulk density

The bulk density and porosity relationships for the samples studied are illustrated in Fig. 5a which shows the reverse relationship characterized by a correlation coefficient of *r* = 0.87 and *r* = 0.67 for facies 1 and 2, respectively. This linear relationship can be due to a similar mineralogical composition for each face, grain shape, packing and sorting; as a result, the pore skeleton should be uniform and homogeneous (Kassab and Teama 2018). The density–porosity ratios in these diagrams are linear and controlled by the following equations.

For facies 1

9 $$\emptyset = 133.41 - 58.65\sigma_{b}$$

For facies 2

10 $$\emptyset = 91.94 - 34.59\sigma_{b}$$

The permeability and bulk density relation is illustrated in Fig. 5b. This is an inverse relationship characterized by a reliable and slightly higher coefficient correlations for the Barail sandstone samples (*r* = 0.58 and 0.68 for Facie1 and 2, respectively). These relationships can be derived from linear equations;

For facies 1

11 $$k = 1E + 8\sigma_{b}^{ - 6.485}$$

For facies 2

12 $$k = 4E + 7\sigma_{b}^{ - 6.485}$$

Permeability and porosity cross-plot (Fig. 5c) shows different positive trends between porosity and permeability characterized by a reliable and intermediate coefficient of correlations for the Barail sandstone samples. (*r* = 0.74 and 0.60 for facie1 and 2, respectively), while some data points are scattered. Exponential regression equations representing such relationships for facies 1 and facies 2.

For facies 1

13 $$k = 27.58\emptyset^{0.09}$$

For facies 2

14 $$k = 20.05\emptyset^{0.10}$$

Figure 5b shows a clear close correlation which illustrates the increase in permeability with the decrease in bulk density of the Barail sandstone samples, while some data points are dispersed. Permeability may therefore be related to bulk density.

The reservoir quality index (RQI) values of the Barail sandstone samples are directly related to the porosity (Fig. 5d). The porosity–RQI relationships of the samples studied are characterized by a correlation coefficient ranging from fair to slightly greater (*r* = 0.35 and 0.09 for facie1 and 2, respectively), while certain data points are dispersed. However, the equations that represent these relationships are:

For facies 1

15 $${\text{RQI}} = 0.054e^{2.574\emptyset }$$

For facies 2

16 $${\text{RQI}} = 0.063e^{1.748\emptyset }$$

The RQI values of Barail sandstone samples are directly related to the permeability shown in Fig. 5e. The permeability and RQI of the studied samples are characterized by a low correlation coefficient (*r* = 0.81 and 0.56 for facies 1 and facies 2, respectively). This means that the reservoir exhibits the high value of heterogeneity. The following equations may be used to represent the relationships:

For facies 1

17 $${\text{RQI}} = 0.05k^{0.003}$$

For facies 2

18 $${\text{RQI}} = 0.05k^{0.003}$$

From the above relationship, we observed that the reservoir quality index RQI is not mainly dependent on permeability. However, rock strength and bulk density have a linear correlation.

(*r* = 0.67 and 0.69 for facies 1 and 2, respectively) between them as compressive strength increases with increases of bulk density (shown in Fig. 5f).

## Petrographic study

**Fig. 6:**
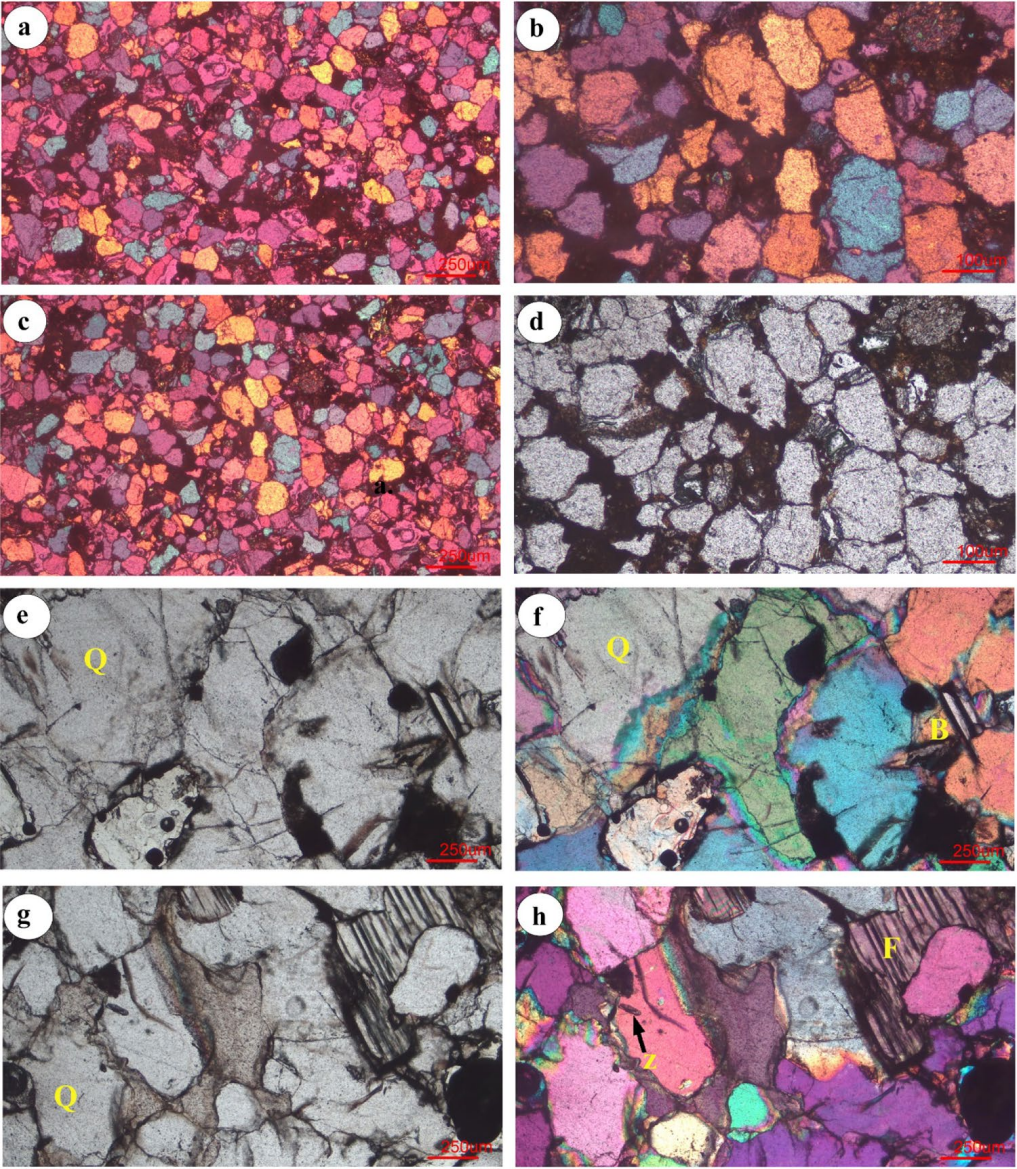
Photomicrographs of the Barail Sandstones showing microcrystalline quartz (Q) of subangular to euhedral shape with suture contact with inclusion of dark-colored minerals. Feldspar (F) are fractured with irregular contact, rock fragments with gneissic product (**b**), Biotite (B) is fracture with a kink bend effect due to compaction (**f**). Iron oxide rim around the quartz grains are also visible (**f**)

**Fig. 7:**
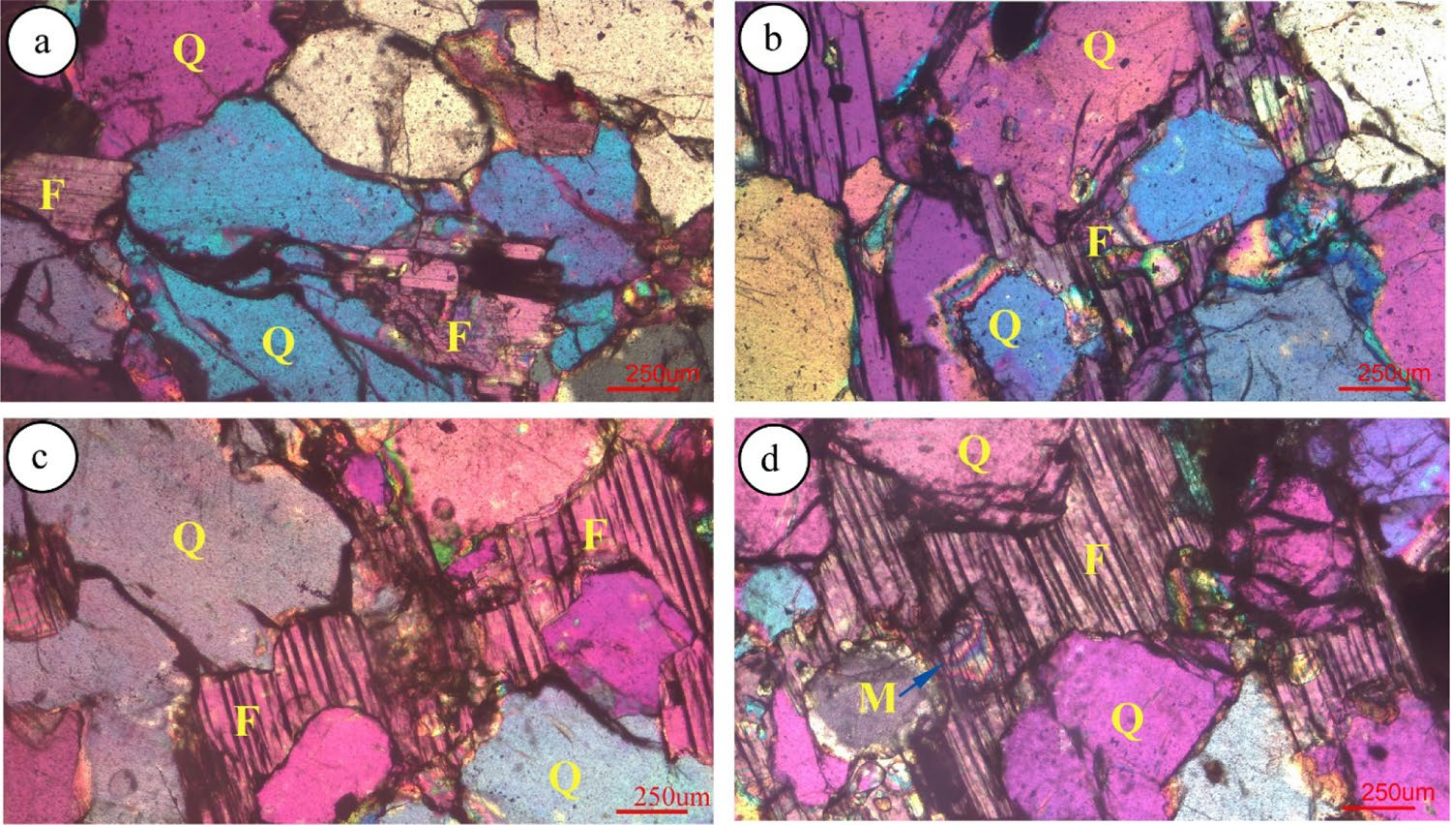
Photomicrographs of Barail Sandstones showing quartz (Q) different types of fractured feldspars (F) with suture contact (**a**–**c**). Muscovite (M) grains is visible (**d**) within the feldspar and dark-colored minerals are found

**Fig. 8:**
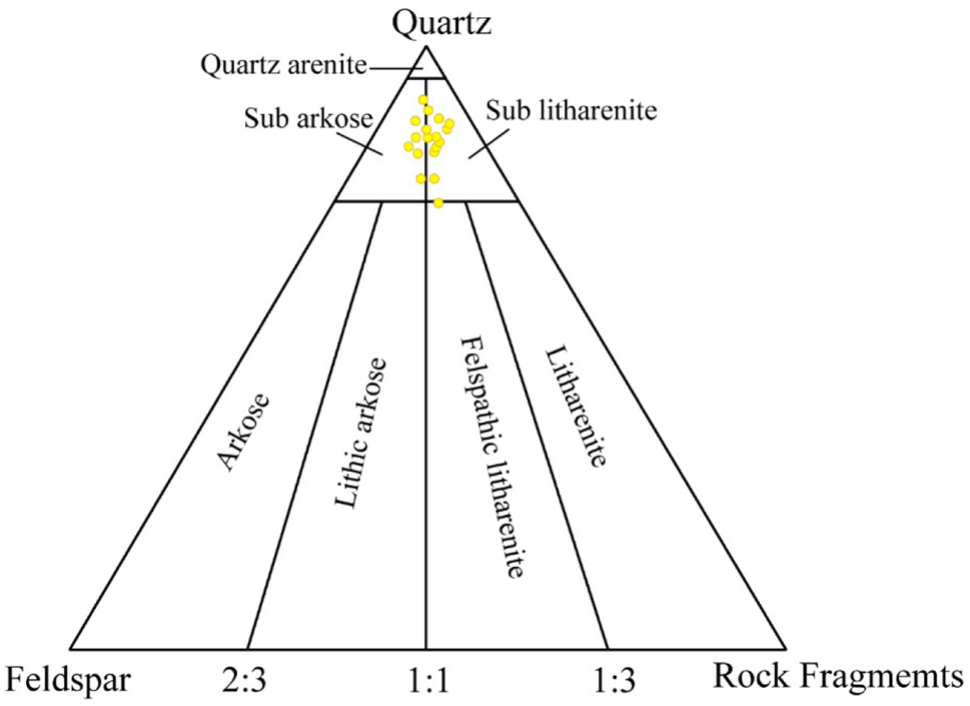
Classification of Barail Sandstones using diagram of (Folk 1974)

**Fig. 9:**
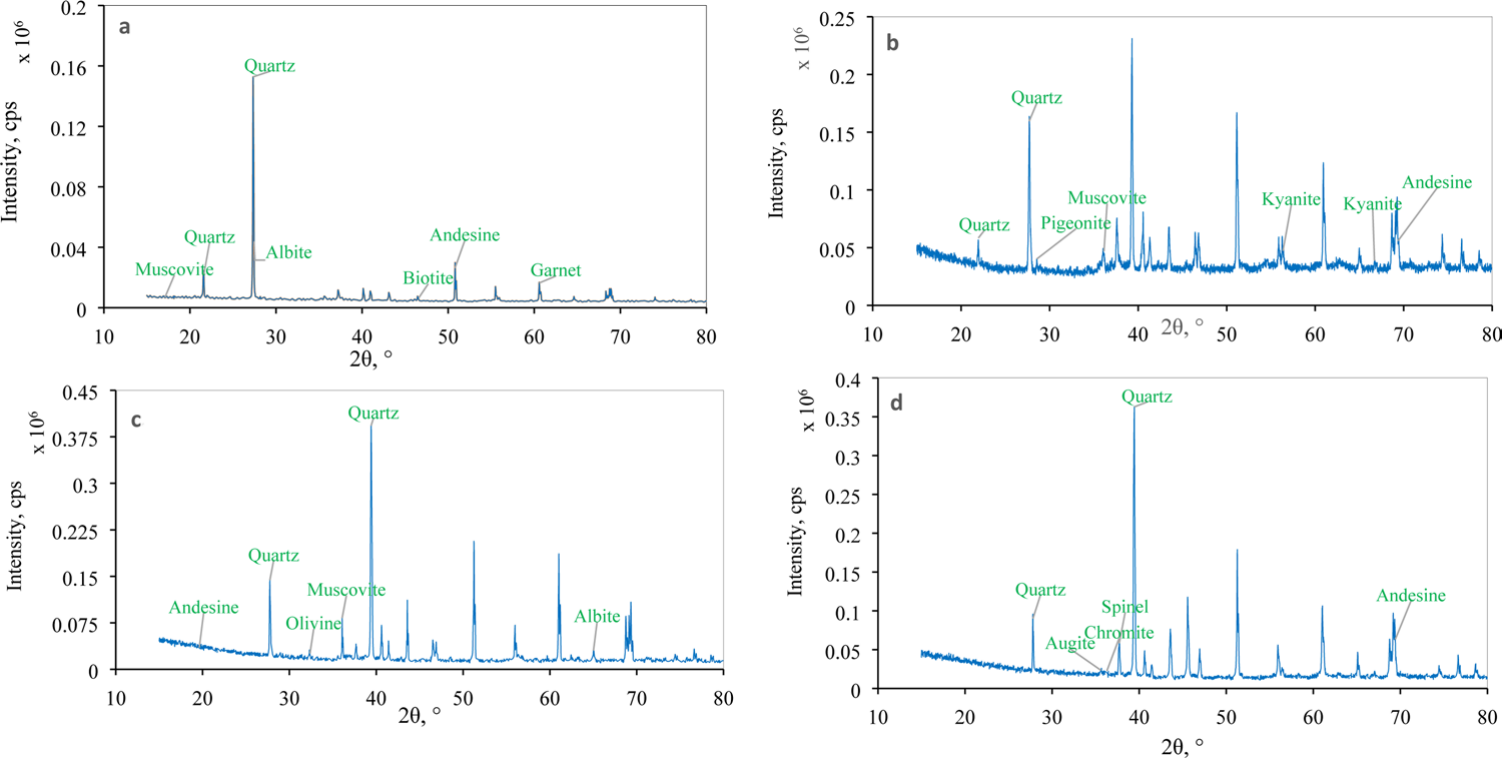
Representative X-ray graphs showing the intensity with different angle and mineral detections with matching for **a** sample 1 **b** sample 3 **c** sample 4 and **d** sample 12

All the thin sections studied consist of quartz and feldspar with a negligible amount of micas (biotite and muscovite) among the rock fragments. Certain dark-colored minerals are found in rock samples (Figs. 6–7). Quartz grains are subrounded to angular with medium to low sphericity and porphyric texture. Most grains are coated with a brown border that could be hematite cement. The majority of large quartz grains are monocrystalline, whereas some are polycrystalline. Quartz grains also characterized by concavo convex, overgrowth and suture contact grain boundary. Zircon grains found inside the quartz at certain locations (as shown in Fig. 6h) except for groundmasses. Feldspar are mostly alkaline feldspar with an irregular shape and some specimens are rich in feldspar plagioclase like albite and andesine (Fig. 7b–d). These display several twin crystals under microscopic observation. A few microcline grains with a perthitic intergrowth containing lamellae which caused the sodium-rich feldspar in the potassium-rich feldspar (Fig. 6g–h). A certain alteration in the cloudy or brown orthoclase feldspar in PPL is visible as a result of chemical alteration. Kaoline clay is visible inside the specimen due to the chemical alteration of feldspar (Fig. 7c). Silica and iron oxide cements are clearly identified in Barail sandstone (Figs. 6–7). The iron oxide cement is in the form of a dark black coating on the detrital grains of quartz and feldspar, as well as on the dispersed groundmass and invading pores. Rock fragments with metamorphic fabrics of gneissic/schistose are found in the thin section (Fig. 6b). Among all varieties of rock fragments, metamorphic types are dominant whereas volcanic products, including

dark-colored minerals, are also notable in some samples (Fig. 7a–d). Surface (areal) calculations using ImageJ software show that quartz, feldspar and rock fragments occupy an average of 87%, 5% and 8% of the total exposure. On the basis of framework grains, Barail sandstone is sub-arkose to sub-arenite using triangular diagram by (Folk 1974) (Fig. 8). XRD analyses support our thin section analyses and show other mineral grains in the rock samples. Minerals identified in the samples are quartz, muscovite, biotite, albite, andesine, zircon, garnet, kyanite, pigeonite, olivine, augite, spinel, ulvo-spinel, chromite, etc. (Fig. 9).

## Discussion

Integration of porosity and permeability with RQI, NPI and FZI used to reservoir quality analysis is popular to quantify the reservoir rocks. However, core plug measurement is still well accepted for porosity and permeability measurement rather than geophysical observation. Porosity and permeability measured in the present study are 16.48% and 132.48 mD (Table 2) which indicate that the Barail sandstone is a good-quality reservoir rock. However, cross-plot between porosity shows strong correlation (Fig. 5b); porosity increases as permeability increases, which also supports good-quality reservoir character. It is obvious that all samples the Barail sandstone do not show linear relation which shows that higher porosity does not always correspond higher permeability or vice versa (Al-Dhafeeri and Nasr-El-Din 2007). RQI in relation to porosity and permeability is used to quantify reservoir quality in some extent and provides a good relationship between petrophysical properties in microlevel for core plug. RQI value of studied sample has less than 1 micron indicative of lesser heterogeneity, inversely indicates a good reservoir quality. Combination of RQI and FZI is used for zonation of different facies in the reservoir. It is believed that the zone of higher RQI and FZI should be a higher potential of permeability. Barail sandstone shows a good agreement with permeability/permeability with RQI and FZI. Low FZI value indicates that the studied Barail sandstone has less heterogeneity and prospective for a good reservoir rock (Al-Dhafeeri and Nasr-El-Din 2007). Rock compressibility is very important for drilling purposes, especially rock strength and stress–strain relationship. Barail sandstone has average rock strength, shear strength, elastic modulus and passion ratio which are 2402.8 kPa, 651.35 kPa, 2574.81 Mpa, and 0.25, respectively (Table 2). This properties indicate that the Barail sandstone is moderately compacted and intermediate strength. Rock strength and bulk density have strong correlation which indicates that rock strength of the rock samples increases as increase of bulk density (Fig. 5f).

The Barail sandstone formation exposed within the Dauki Fault area, and these sediments are believed to be deposited early in the collision of the Indian and Burmese submarine plates and granitoids have been deposited in the active continental margin (Borgohain et al. 2020; Sen et al. 2012). Borgohain et al. (2020) noted that the Barail sandstone from this region is rich in quartz, poor feldspar, has lithic fragments dominated by sedimentary and metamorphic clasts and has been derived from a recycled origin. However, the analysis of the provenance by (Uddin and Lundberg 2004) also suggested the same conclusion and estimated that the Barail sandstone was derived from proto-Himalayan origins. (Borgohain et al. 2020) noted that the Barail sandstone of this region is quartz-rich, feldspar poor, has lithic fragments dominated by sedimentary and metamorphic clasts and was derived from a recycled origin. (Borgohain et al. 2020) also suggested that the Barail sandstone might be derived from recycled origin with subordinate contribution from oceanic arc and active continental marginal setting. The sediments were derived from the uplifted and eroded Himalayan quartz rich (Sen et al. 2012), crystalline felsic terrain and also from eastern orogeny (IBR) along with recycled sediments (Borgohain et al. 2020). Petrographic and XRD analyses showed that the studied rock samples contain mainly quartz (87%) and insignificant amount of feldspar (5%) and rocks are sub-arkose and sub-litharenite (Fig. 6x). Due to medium- to coarse-grained sub-askose to sub-litheranite sandstone indicative of fluvial deposits in the near-shore terrestrial deposits during Oligocene time. Moreover, suture contact between quartz/feldspar grains indicates that Barail sandstone experienced high degree of compaction. The samples contain gneissic lithic material of continental felsic rocks and also contain volcanic product such as augite, spinel and ulvo-spinel (Fig. 9). Petrographic classification (Fig. 8) indicated that samples fall in sub-arkose to sub-litharenite category. This indicates that samples are moderately matured consisting of more rock fragments than feldspar and dominated by individual quartz crystal with porphyritic texture. The plagioclase feldspar (such as albite and andesine) indicates that these grains come from volcanic product of nearby source and weekly a locally reworked pyroclastic rock (Adams et al. 1984). Quartz grains of subrounded, euhedral and moderately sorted also support that the source materials might be proto-Himalayan (Uddin and Lundberg 2004) and IBR origin (Borgohain et al. 2020). Iron oxide rim around quartz grains indicates that samples experienced moderate degree of chemical weathering under arid climatic condition. Moreover, Barail sandstone with moderate maturity exhibits that these rocks have better reservoir quality.

## Conclusion

Grey- to pinkish-colored Barail sandstone in the Surma Basin of Bangladesh is a good-quality reservoir rocks based on its petrophysical and petrographical analyses. Petrographic analyses shows that the studied sandstone samples have 16.48% porosity and 132.48 mD permeability. The sandstones have higher RQI and low FZI values which shows a good prospective reservoir quality. The sandstones is mainly consisted of quartz and an insignificant amount of feldspar and rock fragments. Petrographic analyses showed that the studied rock samples contain mainly quartz (87%) and insignificant amount of feldspar (5%) and rock fragments (7%). Based on volumetric analysis, the sandstones are categorized as sub-arkose to sub-litharenite types. XRD analyses support the thin section analyses and also identified rock fragments within the samples. Feldspars are alkali, but plagioclase feldspar also observed in some thin sections. Rock samples contain gneissic lithic material of continental felsic rocks and also contain volcanic product such as augite, spinel and ulvo-spinel. Iron oxide rim around quartz grains indicates that samples experienced moderate degree of chemical weathering under arid climatic condition. Moreover, Barail sandstone with moderate maturity exhibits that these rocks have better reservoir quality.

## Data Availability

Data are given in Appendix.
